# A Study on the Effects of Embodied and Cognitive Interventions on Adolescents’ Flow Experience and Cognitive Patterns

**DOI:** 10.3390/bs15060768

**Published:** 2025-06-03

**Authors:** Chujie Liang, Jiahao Zhi, Cong Su, Weichun Xue, Zixi Liu, Haosheng Ye

**Affiliations:** Department of Psychology, School of Education, Guangzhou University, Guangzhou 510006, China; 2112408170@e.gzhu.edu.cn (C.L.); 2112308017@e.gzhu.edu.cn (J.Z.); 2112408124@e.gzhu.edu.cn (C.S.); 2112408028@e.gzhu.edu.cn (W.X.)

**Keywords:** flow experience, breathing exercise, adolescents’ mental health, embodied education

## Abstract

This study investigates the effects of embodied (breathing exercises) and cognitive interventions on adolescents’ flow experience and cognition patterns. Using a mixed-methods design, 303 vocational high school students were assigned to three groups: Embodied Task Group (*N* = 108), Cognitive Task Group (*N* = 100), and Mental Health Course Group (*N* = 95). Experiment 1 employed a 3×2 Multivariate Analysis of Covariance (MANCOVA) design to compare flow experience dimensions, while Experiment 2 used Epistemic Network Analysis (ENA) to analyze diary entries. Results showed that the Embodied Task Group outperformed the Cognitive Task Group in “Unambiguous Feedback” (η*_p_*^2^ = 0.01, a small effect) and had higher “Transformation of Time” (η*_p_*^2^ = 0.01, a small effect) than the Mental Health Course Group. ENA revealed that the Embodied Group developed stronger body-environment interaction patterns, shifting cognition pattern from psychological evaluations to dynamic bodily processes over time. Conversely, the Cognitive Task Group maintained event-focused cognition with weaker mind–body integration. Findings highlight breathing exercises’ potential to enhance flow experience through embodied awareness and multisensory processing, offering practical implications for mental health education by promoting embodied learning tasks to foster flow experience.

## 1. Introduction

Current school mental health education primarily adopts collective teaching and cognitive-oriented models. Due to limited class time and inflexible content, students struggle to transfer learned skills into daily contexts amidst hectic academic schedules (Robertson et al., 2023). Against this backdrop, flow experience—a positive psychological state of complete immersion—offers a novel intervention perspective for mental health education, as it can occur in nearly any task or situation (Csikszentmihalyi, 1975). Emerging evidence demonstrates a positive correlation between flow levels and adolescent mental health (Mangialavori et al., 2024). flow experience can arise from cognitive activities or dynamic couplings of physical movement, environmental interaction, and cognitive processing (X. Liu et al., 2023; Montull et al., 2020). From a mental health intervention perspective, studies have shown that breathing exercise can induce flow experience to reduce stress and anxiety (Chin & Kales, 2019). Moreover, breathing exercise represents an efficient self-regulation strategy due to their low implementation barriers and spatiotemporal flexibility (Robertson et al., 2023).

### 1.1. Embodied Education and Mind–Body Interactive Learning

Embodied Education represents an educational philosophy emphasizing “physical engagement”, which requires individuals to cultivate dual awareness of both bodily sensations and environmental interactions, with awareness serving as its core mechanism (Singh & V, 2021). The body plays an important role in this process, simultaneously reflecting somatosensory information and environmental interactions (S. Liu & Ba, 2021). This educational paradigm contrasts with traditional disembodied approaches, which prioritize knowledge transmission and cognitive skill development. Embodied Education emphasizes active physical participation to reduce cognitive load and enhance learning outcomes (Lyu & Deng, 2024). For instance, Yeh et al. (2025) found that increasing physical engagement in classroom settings significantly improves student participation. Another VR-based study demonstrated that embodied learning enhances academic performance, particularly in abstract concept comprehension (Z. Liu & Wang, 2024). Collectively, these findings highlight that knowledge acquisition pathways involve not only abstract symbolic understanding but also concrete bodily experiences (Shapiro & Stolz, 2019).

From the perspectives of Embodied Cognition and Disembodied Cognition, school mental health education models can be categorized into three approaches: physical engagement-based interaction, cognitive interaction, and passive knowledge reception. The physical engagement-based approach focuses on knowledge construction through bodily participation (Singh & V, 2021). The cognitive interaction approach relies on symbolic information feedback mechanisms to facilitate knowledge exchange with the external environment (Beier & Rau, 2022). Conversely, the passive knowledge reception approach does not require active student feedback or comprehension of internal/external information. For example, disembodied education refers to an educational approach where students are not required to actively provide feedback. It centers on conceptual learning rather than direct physical engagement with the environment. In contrast, embodied education emphasizes body–environment coupling. It encourages students to interact with the environment through physical actions and facilitates the development of awareness of bodily and mental sensations as a means of constructing knowledge.

### 1.2. Diary Method and Disembodied Education-Based Interventions

The diary method represents a research approach where participants systematically record their experiences, emotions, and behaviors in naturalistic settings. Cognitive intervention-based diary practices have demonstrated efficacy in improving mental health outcomes (Unterhitzenberger & Lawrence, 2022). Existing studies highlight its role in reinforcing positive cognitions while attenuating negative thought patterns. For example, gratitude journaling has been shown to enhance subjective well-being (K. Yang et al., 2024). Franz et al. (2021) found that diary-based cognitive reappraisal weakens the association between stress and suicidal ideation by disrupting links between negative awareness and behavioral intentions. In adolescent mental health education, diary methods significantly reduce test anxiety (Autayeva, 2024). Despite its widespread application, current research primarily focuses on cognitive interventions, leaving comparative analysis of self-reported texts under embodied vs. cognitive conditions—and trajectory comparisons of longitudinal records—underexplored.

### 1.3. Breathing Exercise and Embodied Education-Based Interventions

Physical engagement-based interventions represent viable approaches for enhancing mental health outcomes (Ku et al., 2025). Embodied interventions strengthen students’ embodied awareness, improving their ability to detect bodily changes, enhance physical fitness, and increase sensitivity to somatosensory fluctuations (Singh & V, 2021). However, not all embodied activities are suitable for educational settings. Most physically intensive flow experience tasks—such as games, psychological interventions, and musical performances—require substantial physical movement and high spatial/equipment demands, making them challenging to implement in routine school settings (Rakei & Bhattacharya, 2024; Dewaele et al., 2019; Hamari et al., 2016).

Breathing exercise, as an embodied intervention method, can also facilitate individuals’ flow experience and improve mental health outcomes (Chin & Kales, 2019). For example, breathing exercise effectively reduces pre-performance anxiety in students (Hu, 2024). Cold exposure (an intervention measure that stimulates the body with cold water to trigger physiological stress responses) combined with breathing exercise can reduce the perceived stress of individuals. A 12-week respiratory intervention significantly improves emotional experiences, academic belongingness, and learning enjoyment among students (Jones & Lee, 2022). In this study, participants engaged in intensive nasal breathing exercise (see Methods for details) to regulate cognitive patterns, filter external distractions, and enhance flow experience (Thissen & Oettingen, 2024).

Although the positive impact of flow experience on adolescents’ mental health has been confirmed (Csikszentmihalyi, 1975; Mangialavori et al., 2024), and both embodied education and cognitive interventions have been proven effective, respectively (Singh & V, 2021; Unterhitzenberger & Lawrence, 2022), several crucial research gaps remain. Existing studies mostly focus on single intervention models, lacking a comparison of the core mechanisms by which embodied interventions (such as breathing exercises) and cognitive interventions promote flow experience. Moreover, there are fundamental differences between the real-time physical regulation mechanism of embodied cognition and the passive information-processing model of traditional cognitive interventions in coping with adversity, yet the specific differences in cognitive strategies between the two remain unexamined (Beier & Rau, 2022). Therefore, this study aims to provide empirical evidence for designing low-threshold embodied tasks (such as breathing exercises) in school mental health education, and to make up for the limitations of traditional cognitive interventions in mind–body regulation. This research investigates the differential effects of embodied vs. disembodied education on adolescents’ flow experience. We operationalized mental health education tasks into two formats: Embodied Cognitive Tasks and Cognitive Tasks. Using diary methods and scales, we analyzed the real-world manifestations of these interventions in daily school settings to explore divergences in cognitive pathways and flow experience between the two modalities.

### 1.4. Research Questions

To investigate the effects of embodied vs. disembodied cognition on adolescents’ flow experience in daily school settings, this study integrated qualitative and quantitative methods guided by Embodied Cognition and Embodied Education theories. In the quantitative component, participants were randomized into three groups: Embodied Task Group (via breathing exercise and Mind–Body Observation Diary), Cognitive Task Group (via Daily Insight Diary practices), and Mental Health Course Group (via traditional classroom instruction). We compared total flow scores and nine flow dimensions scores across daily life contexts. The qualitative component analyzed cognitive-behavioral divergences between the Embodied Task and Cognitive Task groups. Based on this design, three research questions emerged:

RQ1: Do breathing exercise interventions, cognitive interventions, and traditional mental health courses differ significantly in triggering participants’ flow experience?
RQ2: As an embodied cognition-oriented physical exercise method, does repeated breathing exercise enhance participants’ embodied awareness (i.e., increase salience of bodily perceptions)?
RQ3: Do body-led breathing exercise interventions produce distinct cognitive patterns compared to traditional cognitive interventions?

By addressing these research questions, this study aims to inform targeted and practical intervention strategies for adolescent mental health education, optimizing educational outcomes through evidence-based practices.

Additionally, this research has been preregistered at https://osf.io/ (https://doi.org/10.17605/OSF.IO/T83AP; accessed on 18 January 2025).

## 2. Materials and Methods

### 2.1. Experiment 1

#### 2.1.1. Participants

Six classes were randomly selected from a vocational high school in Foshan City for this study: two classes received breathing exercise interventions (Embodied Task Group), two engaged in cognitive interventions (Cognitive Task Group), and two participated in mental health courses (Mental Health Course Group). Participant demographics were as follows:

Embodied Task Group: 108 students (*M* = 15.67, *SD* = 0.38; 32 males)

Cognitive Task Group: 100 students (*M* = 15.57, *SD* = 0.34; 55 males)

Mental Health Course Group: 95 students (*M* = 15.54, *SD* = 0.38; 41 males)

Participants received CNY 50 worth of pastries and beverages as compensation. This study was approved by the Ethics Review Committee (IRB), and all participants provided informed consent prior to data collection.

#### 2.1.2. Design

Experiment 1 employed a 3 (Intervention Type: Respiratory/Cognitive/Traditional Mental Health Course) × 2 (Measurement Time: Pretest/Posttest) two-factor mixed design. The dependent variables included nine flow experience dimensions (Challenge-skill balance, Action-awareness merging, Clear goals, Unambiguous feedback, Total concentration, Sense of control, Loss of self-consciousness, Transformation of time, Enjoyment) and total scores measured by the Simplified State Fluency Scale (Lee-Shi & Ley, 2022).

#### 2.1.3. Procedure

**Figure 1:**
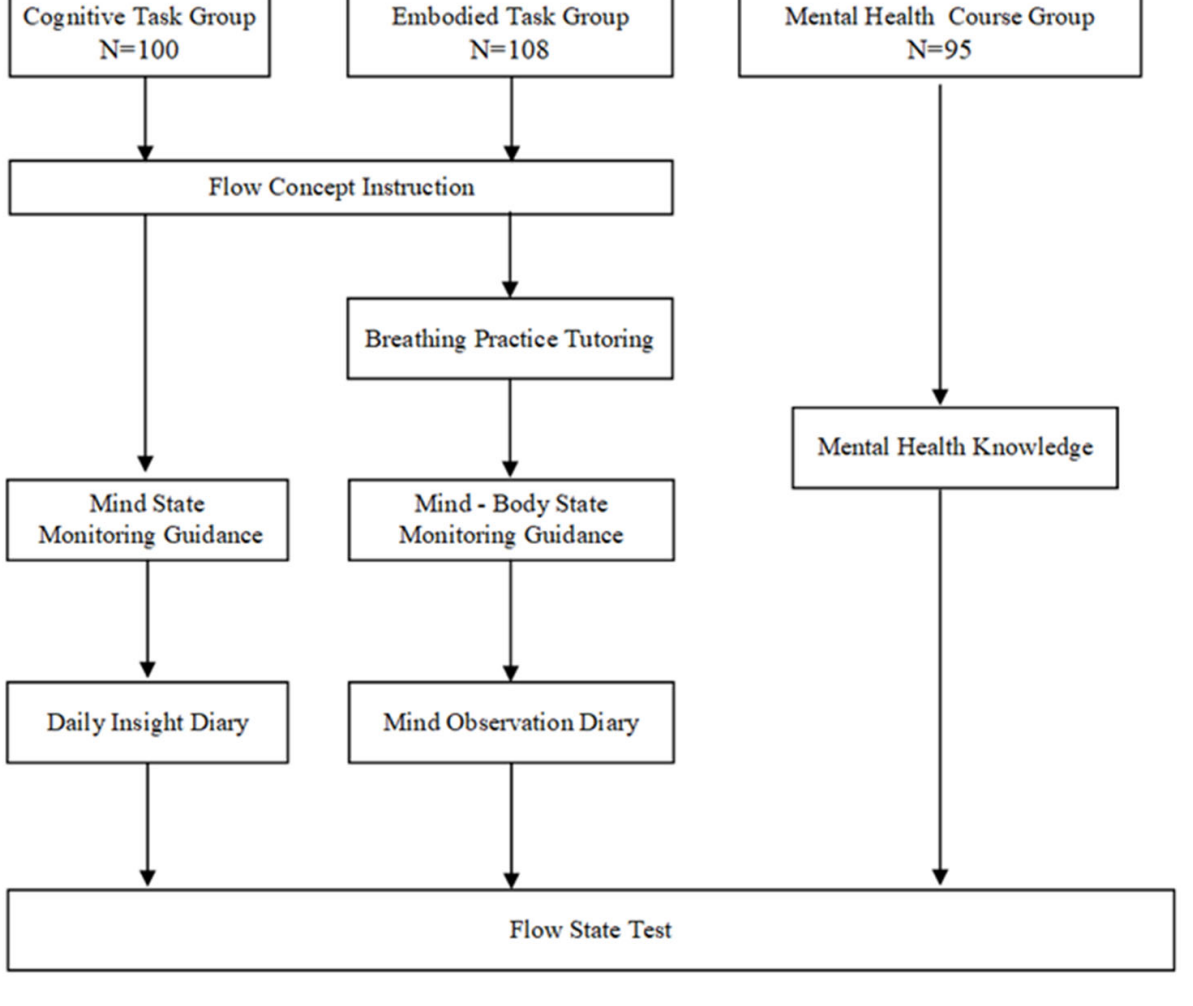
Schematic diagram of the intervention process.

After randomly selecting six classes from the first grade of senior high school in the school, the six classes were randomly assigned to three experimental conditions (Cognitive Task Group/Embodied Task Group/Mental Health Course Group). We implemented an intervention program lasting for 20 days for different groups (Lehmann & Murakami, 2024). All participants, in addition to completing the tasks they were assigned to, were required to complete a measurement of the flow experience once a week (Figure 1). The specific course materials can be found in the Supplementary Materials.

Cognitive Task Group

(1) Flow Concept Instruction: The experimenter introduced the concept of flow experience to the participants in a mental health education class and elaborated on to the flow theory and the conditions that trigger flow experience. These conditions mean being fully engaged in various activities.
(2) Mind State Monitoring Guidance: The experimenter advised participants to pay cognition to their daily state changes. At the same time, participants need to ensure that they maintain awareness of their daily state by writing diaries.
(3) Daily Insight Diary: The Daily Insight Diary is a journaling activity where participants are instructed to monitor and document their daily state changes, including thoughts, emotions, and behaviors, during their regular studies and daily lives. This diary aims to cultivate participants’ self-awareness and promote a reflective understanding of their daily experiences, helping them maintain consistent perception of their internal and external states over time. Ultimately, each participant will complete a 20-day diary.

Embodied Task Group

(1) Flow Concept Instruction: Similar to the cognitive task group, the experimenter explained the concept of flow experience to the participants in a mental health education class.
(2) Breathing Practice Tutoring: The experimenter first explained the breathing method to each participant. Once all participants had understood the method, the breathing exercise guidance began. The audio guidance for the breathing exercise was as follows: Return to your body and focus on your breath. Inhale and exhale through your nose. Continuously exhale the air from your body, and when it becomes unbearable, inhale. Repeat this process until the end of the exercise (H. Yang, 2023). The duration of the exercise session is 10 min.
(3) Mind–Body State Monitoring Guidance: During the breathing exercise, the experimenter instructed the participants to observe daily changes in their mind–body states. They were asked to write diaries to identify specific changes brought about by the above methods and describe their own states. This descriptive approach aims to reduce the interference of external events and distractions, enabling participants to directly observe changes in their mind–body states.
(4) Mind–Body Observation Diary: The Mind–Body Observation Diary, an extension of the Daily Insight Diary, is designed to minimize the influence of external events and distractions. It allows participants to focus on and directly observe how breath-focused practice affects their mind–body connection in daily activities. Participants are required to describe these experiences in detail both immediately after the breathing exercises and throughout the day as they integrate the breathing practice into their routines. Finally, each participant will compile a 20-day diary.

Mental Health Course Group

The teaching content of this course mainly focuses on positive emotions and the analysis of common emotional confusions among adolescents, covering four key aspects: understanding basic emotions (joy, anger, sorrow, and happiness), building self-confidence, cultivating a sense of gratitude, and recognizing the psychological development patterns of adolescents. The goal of this course is to enhance students’ mental health knowledge and deepen their understanding of adolescent psychological characteristics.

### 2.2. Experiment 2

#### 2.2.1. Participants

Participants in Experiment 2 were identical to those in Experiment 1’s Cognitive Task Group (*N* = 100) and Embodied Task Group (*N* = 108).

#### 2.2.2. Design

To address RQ2, this study employed a single-factor 3-level (Intervention Phase: Stage1/Stage2/Stage3) within-subjects design. Using Epistemic Network Analysis (ENA), we examined 20-day longitudinal changes in cognitive network structures among participants in the Embodied Task Group.

For RQ3, a single-factor 2-level (Embodied Task Group/Cognitive Task Group) between-subjects design was utilized. Cognitive network differences between the two groups were analyzed via ENA to explore divergences in cognitive patterns allocation and cognitive patterns.

#### 2.2.3. Procedure

The procedures for Experiment 2 aligned with those of Experiment 1’s Embodied and Cognitive Task Groups, conducted synchronously. Participants engaged in both experiments concurrently, providing distinct data dimensions for each study under identical task experiences. Every night before bedtime, participants completed paper-and-pencil diaries with 3–4 questions.

Embodied Task Group answered four questions:

(a) Physical and mental experiences immediately following breathing exercises.
(b) Bodily and emotional states, as well as response processes, during favorable and challenging situations.
(c) Environmental perceptions across different contexts.
(d) Physical and mental experiences during activities like studying, focused work, and play.

Cognitive Task Group answered the last three questions (excluding Question a).

During data collection, returned diaries underwent screening based on two criteria:

(a) Incomplete or non-compliant responses;
(b) Substantial daily content repetition.

After screening, a total of 163 valid diaries were collected, including 90 diaries from the Embodied Task Group and 73 diaries from the Cognitive Task Group.

#### 2.2.4. Statistical Analysis

In Experiment 1, data analysis was performed using IBM SPSS Statistics 27.0. This study employed Multivariate Analysis of Covariance (MANCOVA) to examine the effects of two independent variables—intervention method and pre-post measurement—on scores across nine dimensions and the total score of the Short Flow State Scale.

In Experiment 2, qualitative data coding was initially performed in Microsoft Excel 2021, which was subsequently analyzed using Epistemic Network Analysis (ENA) via the ENA Web Tool (v1.7.0; Bowman et al., 2021).

#### 2.2.5. Data Coding

A post hoc coding approach was employed, guided by the study’s theoretical framework and research objectives. A problem-specific coding scheme was developed for each research question through inductive analysis of participants’ diary entries. Inter-rater reliability was established via a three-stage coding process. First, 11% (*n* = 18) of the total diaries were randomly selected for pilot coding (Chen et al., 2022). Three researchers independently performed the coding tasks based on the initial coding scheme. After that, Fleiss’ Kappa coefficient was used for inter-rater reliability assessment. When coding discrepancies occurred, researchers elaborated on their understanding and judgment criteria for controversial codes from a professional perspective. They engaged in discussions around coding rules and standards to refine and improve the operational definitions of the codes. During this process, the research team conducted three intensive discussion sessions to reach a consensus on unresolved discrepancies and newly identified issues. The final inter-rater reliability analysis, using Fleiss’ Kappa coefficient (*κ* = 0.78), indicated substantial agreement according to Landis and Koch’s (1977) guidelines. The detailed operational definitions can be accessed at the website https://doi.org/10.17605/OSF.IO/T83AP. The meanings of the codes for each question are as follows:

Question a:

Focus on Body (FB): Participants directly observe and describe their bodily sensations, physiological responses, movements, or postures. Examples: Muscle tension, heart rate changes, postural adjustments.

Focus on Psychological Experiences and Feelings (FPEF): Participants describe their internal psychological experiences and emotions, including specific emotional states, intensity, duration, and subjective preferences/aversions. Examples: happiness, sadness, anxiety.

Focus on the Process of Physical and Mental Changes (FPPMC): Participants document how their physical and mental states evolve over time, including individual changes and bidirectional interactions. Examples: How physical fatigue influences emotional states or how emotional shifts trigger bodily reactions.

Question b:

Coping Process in Adverse Situations (CPAS): Participants describe strategies and behavioral processes employed during challenging situations, including coping tactics, implementation frequency, and decision-making patterns. Examples: Proactive problem-solving, seeking social support.

Physical and Mental States in Adverse Situations (PMSAS): Participants record real-time bodily and emotional responses during adversity, including negative emotion intensity/duration and mind–body interactions. Examples: How stress increases physical tension or how physical fatigue exacerbates psychological strain.

Question c:

Mark Focus Events (MFE): Participants identify one or more focal events for documentation, including objective circumstances and their reactions. Examples: Event timing, location, involved individuals.

Extensive Situation Experience Feedback (ESEF): Participants report feelings or overall evaluations of scenarios in learning, work, socializing, leisure, or daily life. Examples: Comfort levels, fatigue.

Physical and Mental States in the Physical Environment (PMSPE): Participants record bodily sensations, physiological responses, and associated psychological states in indoor or outdoor settings. Examples: Feeling claustrophobic indoors.

Question d:

Outcome Consciousness (OC): Participants describe subjective feelings about specific events/activities/phases and conclusions drawn therefrom. Examples: Satisfaction with task completion outcomes.

Process Consciousness (PC): Participants provide multidimensional descriptions (e.g., physical sensations, emotional experiences, cognitive processes) reflecting comprehensive mind–body evolution across space and time. Examples: Transition from tension to relaxation during an activity.

Based on coding results from the Embodied Task Group’s responses to “physical and mental experiences immediately following breathing exercises”, we compared the developmental changes in the group’s cognitive network over time. The 20-day period was divided into three phases: 7-day (Stage 1), 7-day (Stage 2), and 6-day (Stage 3).

ENA is a technique for modeling the structure of connections in data. ENA assumes (1) that it is possible to systematically identify a set of meaningful features in the data (Codes); (2) that the data have local structure (conversations); and (3) that an important feature of the data is the way that Codes are connected to one another within conversations (Bowman et al., 2021). In a diary transcript, each answer to a day might be a unique conversation, or in a collection of documents, each document or section of a document. ENA models the connections among Codes by quantifying the co-occurrence of Codes within conversations, producing a weighted network of co-occurrences, along with associated visualizations for each unit of analysis in the data. Critically, ENA analyzes all of the networks simultaneously, resulting in a set of networks that can be compared both visually and statistically. We defined the units of analysis as all lines of data associated with a single value of intervention mode subsetted by participants.

The ENA algorithm uses a moving window to construct a network model for each line in the data, showing how codes in the current line are connected to codes that occur previously, defined as 7 lines (each line plus the 6 previous lines) within a given conversation (Siebert-Evenstone et al., 2017). The 7-line window length was selected as it effectively captures immediate relationships between cognitive elements in the 20-day intervention data, without including overly distant information. The resulting networks are aggregated for all lines for each unit of analysis in the model. In this model, we aggregated networks using a binary summation in which the networks for a given line reflect the presence or absence of the co-occurrence of each pair of codes.

Our ENA model included the following codes: CPAS, PMSAS, MFE, ESEF, PMSPE, OC, and PC. The ENA model normalized the networks for all units of analysis before they were subjected to a dimensional reduction, which accounts for the fact that different units of analysis may have different numbers of coded lines in the data. For the dimensional reduction, we used a singular value decomposition, which produces orthogonal dimensions that maximize the variance explained by each dimension (Bowman et al., 2021).

ENA can be used to compare units of analysis in terms of their plotted point positions, individual networks, mean plotted point positions, and mean networks, which average the connection weights across individual networks. Networks may also be compared using network difference graphs. These graphs are calculated by subtracting the weight of each connection in one network from the corresponding connections in another.

To test for differences, we applied a Mann–Whitney test to the location of points in the projected ENA space.

## 3. Results

A significant difference in gender distribution among the three classes (*χ*^2^(2) = 15.67, *p* < 0.05). However, after incorporating gender as a covariate into the MANCOVA, the main effects of the gender covariate were not significant (*p* > 0.05). Table 1 reports the dimensions with significant differences in MANCOVA.

In the Unambiguous Feedback dimension, neither the pre-post test main effect (*F*(1, 439) = 2.38, *p* = 0.12, $\eta_{p}^{2}$ = 0.01) nor the intervention method main effect (*F*(2, 439) = 0.37, *p* = 0.69, $\eta_{p}^{2}$ = 0.00) reached significance, but the intervention method × pre-post test interaction was significant (*F*(2, 439) = 3.19, *p* = 0.04, $\eta_{p}^{2}$ = 0.01), though the effect size remained small ($\eta_{p}^{2}$ = 0.01, representing a 1% variance explained by the intervention-time interaction).

In the Transformation of Time dimension, the pre-post test main effect was non-significant (*F*(1, 439) = 0.67, *p* = 0.41, $\eta_{p}^{2}$ = 0.00), but the intervention method main effect was significant (*F*(2, 439) = 3.20, *p* = 0.04, $\eta_{p}^{2}$ = 0.01), indicating a small but statistically meaningful group difference in time perception; however, the intervention method × pre-post test interaction was non-significant (*F*(2, 439) = 1.10, *p* = 0.33, $\eta_{p}^{2}$ = 0.00).

For the total score and the remaining seven dimensions, all pre-post test main effects (*p* > 0.05), intervention method main effects (*p* > 0.05), and intervention method × pre-post test interactions (*p* > 0.05) were statistically non-significant.

**Figure 2:**
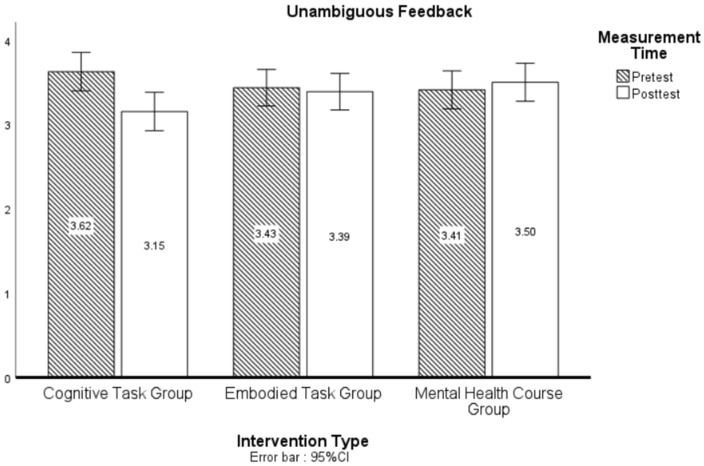
Simple effect analysis of pre-post test, intervention on transformation of time.

Figure 2 presents the outcomes of the simple effect analysis examining the influence of the pre-post test and the intervention method on the Unambiguous Feedback dimension. A significant difference was observed between the Embodied Task Group and the Mental Health Course Group under the posttest condition (AD = 0.43, *p* = 0.02), while no significant differences were detected in all other comparisons (*p* > 0.05).

**Figure 3:**
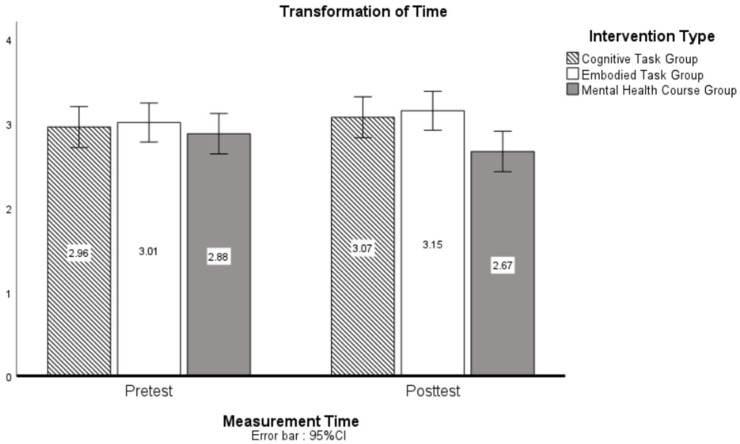
LSD post hoc analysis of intervention on unambiguous feedback.

Figure 3 shows least significant difference (LSD) post hoc results for the intervention method main effect in the Transformation of Time dimension. Findings indicated a statistically significant difference between the Mental Health Course Group and the Mental Health Group (AD = 0.27, *p* = 0.02), whereas no significant intergroup variations were detected in all other comparisons (*p* > 0.05).

For RQ 2, as shown in Figure 2, the first dimension of the ENA comparison graph, that is, the X-axis (SVD1), accounted for 79.1% of the data variance, and the second dimension, that is, the Y-axis (SVD2), accounted for 15.7% of the data variance. The differences between stages 1 and 2 and between stages 2 and 3 were not significant in the X-axis (SVD1) and Y-axis (SVD2). But the differences between Stage 1 and Stage 3 are significant. Specifically, along the X axis (SVD1), a Mann–Whitney test showed that Stage 1 (Mdn = 0, *N* = 90) was statistically significantly different at the alpha = 0.05 level from Stage 3 (Mdn = 0, N = 90 U = 4793.50, p = 0.03, r = −0.18). Along the Y axis (SVD2), a Mann–Whitney test showed that Stage 1 (Mdn = 0.06, *N* = 90) was not statistically significantly different at the alpha = 0.05 level from Stage 3 (Mdn = 0, N = 90 U = 3926.50, p = 0.72, r = 0.03).

**Figure 4:**
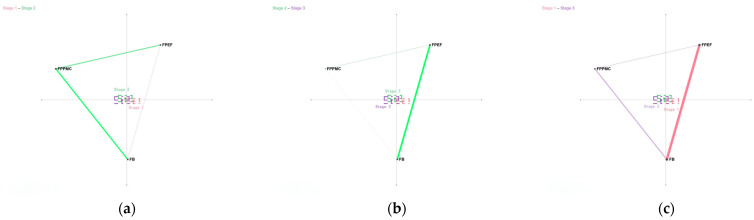
(**a**) Comparison between stage 1 and stage 2; (**b**) comparison between stage 2 and stage 3; (**c**) comparison between stage 1 and stage 3. Note: The green line segment represents that the connection in Stage 1 is stronger in this comparison; the pink line segment represents that the connection in Stage 2 is stronger in this comparison; and the purple line segment represents that the connection in Stage 3 is stronger in this comparison. For the same color, the thicker the line segment and the darker the color, the stronger the connection.

From the comparison graph (Figure 4), co-occurrence between FB and FPEF was greater in Stage 1 than Stage 3 (Figure 4c), whereas co-occurrence between FB and FPPMC increased in Stage 3 compared to Stage 1. During the initial intervention phase (Stage 1), individuals simultaneously focused on FB and FPEF. However, in the later intervention phase (Stage 3), this dual focus shifted: participants reduced awareness to FPEF and prioritized documentation of FPPMC.

**Figure 5:**
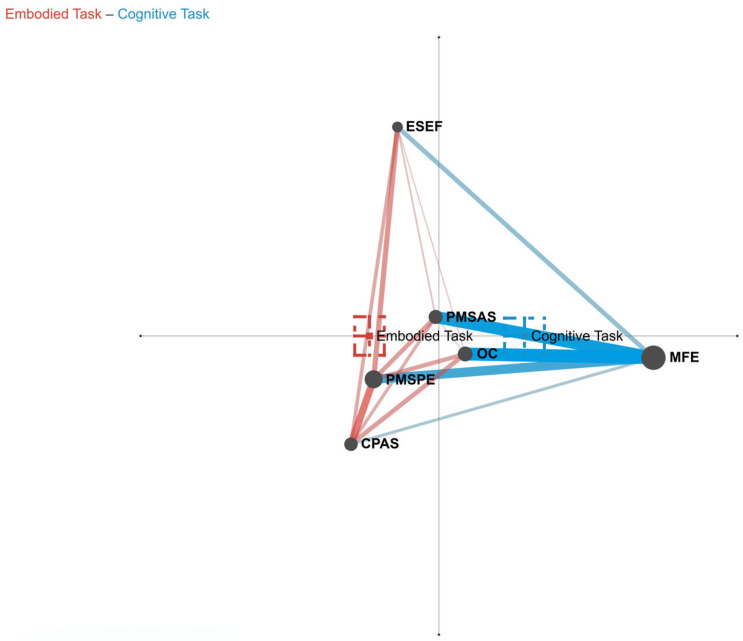
Comparison plot of Cognitive Task Group and Embodied Task Group. Note: The red line segment represents that the connection of the Embodied Task Group is stronger in this comparison; the blue line segment represents that the connection of the Cognitive Task Group is stronger in this comparison. For line segments of the same color, the thicker the line segment and the darker the color, the stronger the connection.

For RQ 3, as shown in Figure 5, the first dimension of the ENA comparison graph is the X-axis (SVD1), and this dimension accounts for 36.1% of the data variance and the second dimension is the Y-axis (SVD2), which accounts for 21.9% of the data variance. Along the X axis (SVD1), a Mann–Whitney test showed that Embodied Task Group (Mdn = −0.24, *N* = 90) was statistically significantly different at the alpha = 0.05 level from Cognitive Task Group (Mdn = 0.30, *N* = 73, *U* = 607.50, *p* = 0.00, *r* = 0.82). Along the Y axis (SVD2), a Mann–Whitney test showed that Embodied Task Group (Mdn = 0.05, *N* = 90) was not statistically significantly different at the alpha = 0.05 level from Cognitive Task Group (Mdn = 0.02, N = 73, U = 3188.00, p = 0.75, r = 0.03).

As shown in the comparison graph (Figure 5), co-occurrences in the Embodied Task Group cluster primarily along the negative half of the X-axis, characterized by dyadic connections between PMSPE and CPAS, as well as their respective links to ESEF, PMSAS, and OC. By contrast, co-occurrences in the Cognitive Task Group concentrate on the positive X-axis, manifesting as dyadic connections between MFE and ESEF, PMSAS, OC, PMSPE, and CPAS.

## 4. Discussion

### 4.1. The Influence of Embodied Interventions on Flow Experience

For RQ1, Experiment 1 was designed to explore the effects of the Embodied Task Group, Cognitive Task Group, and Mental Health Course Group on participants’ flow experience. Statistically significant differences were detected in the “Unambiguous Feedback” and “Transformation of Time” dimensions.

In the “Unambiguous Feedback” dimension, the Embodied Task Group showed better performance than the Cognitive Task Group. It is likely that the breathing exercises in the Embodied Task Group provided direct physical sensations and multisensory information, potentially alleviating cognitive fatigue and enhancing the flow experience in this dimension (Lyu & Deng, 2024). In contrast, the Cognitive Task Group relied on abstract symbolic feedback in a disembodied education format. This might have led to a single-mode of class participation, intensifying cognitive fatigue and reducing the group’s flow experience (Lyu & Deng, 2024). “Unambiguous Feedback” requires participants to clearly perceive and judge their states (W. N. Liu, 2010). The cognitive patterns in the Cognitive Task Group might have hindered participants from effectively recognizing their states and learning conditions, which was not conducive to self-evaluation. On the contrary, the cognitive patterns in the Embodied Task Group enabled participants to be more aware of state changes, facilitating self-awareness and self-regulation. These differences might contribute to the Embodied Task Group’s relatively better performance in the “Unambiguous Feedback” dimension.

Regarding the “Transformation of Time” dimension, the Embodied Task Group achieved a significantly higher score than the Mental Health Course Group. This finding is somewhat consistent with previous research indicating that time perception distortion is related to physiological and psychological mechanisms (Tan et al., 2024). During the breathing exercises, the Embodied Task Group’s focus on respiratory sensations may have modulated their temporal awareness (Rakei & Bhattacharya, 2024).

However, it is important to note that the effect sizes for these significant findings were small ($\eta_{p}^{2}$ = 0.01). This is likely due to two main reasons. First, in the students’ daily campus life, the 10 min breathing exercise was a relatively short addition compared to the whole day. When to add breathing exercises in the daily campus life depends on whether the participants develop the awareness of regulation during the breathing and diary exercises. This is a gradual process. Second, the flow experience scale measured the overall daily flow, not just the impact of breathing exercise alone. Yet the study enables us to better observe the process by which the participants develop the awareness of autonomous regulation in the initial stage of the exercise, which is of great significance. At the same time, future long-term studies can further explore the in-depth impacts of both the immediate and long-term effects on the participants’ flow experience and cognitive development.

In summary, the Embodied Task Group differed significantly from disembodied task groups in the two dimensions. Embodied tasks seem to enhance flow experience in “Unambiguous Feedback” and “Transformation of Time”.

### 4.2. The Impact of Breathing Exercises on the Sensitivity of Bodily Perception

Regarding RQ2, in Stage 1, Experiment 2 indicates that participants focused on both bodily sensations and psychological experiences simultaneously. By Stage 3, however, their awareness shifted from psychological aspects to emphasizing bodily sensations and their dynamic interactions with mental states. This shift suggests that respiratory interventions, based on embodied cognition principles, can enhance participants’ embodied awareness through repeated practice. Bodily perception consists of Interoceptive Awareness (IAw), the conscious detection of bodily sensations, and Interoceptive Accuracy (IAc), the ability to accurately perceive sensory changes (Ceunen et al., 2013). In this study, guiding participants to focus on respiratory processes increased their IAw. This shifted their awareness from psychological evaluations to present-moment bodily experiences, which is consistent with previous research showing that breathing exercises can enhance interoceptive awareness (Farb et al., 2013; D’Antoni et al., 2022).

Breathing exercise not only improves IAc in breathing exercise (Daubenmier et al., 2013) but also helps form a stable embodied cognitive pattern. This pattern enables individuals to more readily interpret their bodily states in daily life. These findings have important implications for adolescent development. Adolescents are in a phase of rapid cognitive development and benefit from diverse learning methods for comprehensive growth. Embodied tasks, like the breathing exercises used in this research, provide a different learning experience compared to traditional cognitive intervention. Through physical participation, students can gain a deeper understanding of knowledge and themselves, potentially enhancing learning immersion and effectiveness (Davis et al., 2023). Adolescents frequently face stress and challenges and need effective coping strategies to maintain good mental health. The increased embodied awareness resulting from these interventions may help them better perceive and regulate their physical and emotional states. This could enhance their stress-coping abilities. Therefore, embodied interventions such as the breathing exercises studied here may have practical value in adolescent mental health education. Further research could explore how to effectively integrate such interventions into educational settings to promote both academic development and emotional well-being among adolescents.

### 4.3. Differences Between Embodied and Disembodied Interventions

When addressing RQ3, a notable divergence in cognition patterns was observed between the Embodied Task Group and the Cognitive Task Group. The Embodied Task Group’s approach emphasized body-environment interactions and bodily sensations, while the Cognitive Task Group concentrated more on cognition-related aspects.

ENA dimensional analysis indicated that the X-axis (SVD1), which accounted for 36.1% of the variance, might represent a crucial difference between body-oriented and cognition-oriented processing. In the Embodied Task Group, negative weights on SVD1 were associated with real-time experiences dominated by the body. Conversely, in the Cognitive Task Group, positive weights on SVD1 were related to event-focused reflection. This suggests that the Embodied Task Group’s cognition patterns were shaped by immediate body–emotion interactions, while the Cognitive Task Group promoted the formation of logical cognitive structures through event analysis.

In the Embodied Task Group, body-centered breathing exercises and mind–body state monitoring guided participants to pay awareness to real-time bodily reactions (i.e., PMSPE) and processes for coping with adversity (i.e., CPAS). The co-occurrence network of this group clustered mainly along the negative X-axis. The strong connection between PMSPE and CPAS, along with their links to ESEF, Physical and PMSAS, and OC, reflected the influence of bodily states on emotional responses and environmental evaluations. This pattern implies that breathing exercises might integrate bodily, emotional, and environmental information by enhancing proprioception.

For the Cognitive Task Group, although cognitive activities were predominant, participants still took in information from body–environment interactions during cognitive processing. Their co-occurrence network formed a radiating pattern centered around MFE. The diary content, which focused on MFE and OC, caused the co-occurrence network to skew towards the positive X-axis. This structure indicated an event-driven bodily information integration pattern, where participants passively included bodily sensations as event attributes rather than actively regulating cognitive processes through bodily perception.

A comparative analysis of network connection patterns under adversity revealed that in the Cognitive Task Group, the co-occurrence of PMSAS and CPAS with MFE was weaker compared to the Embodied Task Group. In the face of adversity, Cognitive Task Group members first focused on the event (MFE) and then passively associated PMSAS and CPAS with it. In contrast, in the Embodied Task Group, PMSAS was connected to both CPAS and PMSPE. This connection suggests that participants in this group maintained awareness of their mind–body states during adversity-coping processes in both general physical environments and adverse situations. Moreover, under adversity, CPAS in the Embodied Task Group was connected to ESEF and OC, indicating that the coping processes integrated situational experience information and had an impact on the perceived outcomes after the adversity.

In summary, breathing exercises in the Embodied Task Group appeared to strengthen proprioception and integrate information related to the body, environment, and emotion. On the other hand, the Cognitive Task Group regarded bodily sensations as mere event attributes. This difference highlights the distinct mechanisms between embodied and cognitive interventions. Embodied interventions rely on dynamic body–environment interactions, while cognitive interventions depend on direct information sources related to focal events.

It should be noted that some findings, although not directly related to RQ3, are still relevant. For example, in the Unambiguous Feedback dimension, the Cognitive Task Group had lower scores than the Embodied Task Group, and in the Transformation of Time dimension, the Embodied Task Group scored higher than the Mental Health Course Group. Additionally, the Embodied Task Group demonstrated enhanced embodied awareness and a shift towards process-oriented cognitive integration. These aspects might contribute to the flow experience and the development of a systemic cognitive framework that synthesizes bodily, environmental, and event-related information (Beier & Rau, 2022; Xu et al., 2025).

### 4.4. Practical Implications

Embodied interaction learning, cognitive interaction learning, and knowledge-input learning vary in flow experience activation mechanisms. Embodied interaction learning forms bodily perception-based cognition via dynamic body–environment coupling. Multimodal information integration in this process might enhance learning engagement. Compared with cognitive interaction learning, which relies on cognitive-operation feedback, embodied interaction learning may mitigate flow experience attenuation caused by cognitive resource depletion through real-time bodily feedback loops. Knowledge-input learning, lacking physical engagement and immediate feedback, shows differences in flow experience over time compared to embodied interaction learning. These findings imply that educational practices could prioritize designing embodied learning tasks, as they might foster immersive states through body–environment interactions (Davis et al., 2023).

ENA results indicated three differences between the Embodied Task Group and the Cognitive Task Group. First, for cognitive conclusion-generation mechanisms, the Embodied Task Group’s network integrated body and environment, with conclusions from real-time processing of embodied information on mind–body states and environmental interactions. The Cognitive Task Group’s cognition-centered network generated conclusions mainly through selective awareness to past focal events (Montull et al., 2020). This difference stemmed from distinct information-input modes: multimodal bodily feedback in the Embodied Task Group versus unimodal cognitive operations in the Cognitive Task Group. Embodied interventions might disrupt cognitive inertia and increase awareness to diverse information.

Second, cognitive patterns differed. The Embodied Task Group initially focused on bodily sensations and developed body–environment cognitive patterns over time. The Cognitive Task Group maintained focus on external events, which might limit awareness of unexpected environmental cues (Gable et al., 2016). Embodied interventions might expand cognitive openness through bidirectional feedback, offering more problem-solving opportunities for adolescents. Cognitive interventions’ focal-event mode might cause cognitive narrowing, restricting adaptability to complex environments (Han, 2020).

Third, adversity-coping mechanisms differed. The Embodied Task Group attended to mind–body states and actively used bodily and environmental information to respond to adversity. The Cognitive Task Group focused on the adversity event itself, passively integrating mind–body states into event representations without active coping actions.

### 4.5. Methodological Contributions

This study utilized the Short Flow State Scale to quantify multidimensional characteristics of flow experience and innovatively employed ENA to reveal dynamic cognitive structural changes under different interventions. The scale achieved quantitative evaluation comparability across nine theoretical dimensions (W. N. Liu, 2010). Transcending traditional linear analysis frameworks, ENA uncovered differentiated features between embodied and cognitive interventions in cognitive patterns and cognitive element coupling mechanisms through co-occurrence network modeling.

### 4.6. Limitations and Future Directions

First, this study was limited by its 20-day intervention cycle, failing to examine the long-term effects of different intervention paradigms on adolescents’ self-regulation strategy formation. Future studies could extend follow-up periods to three years or more, combining Experience sampling method with behavioral observation scales to systematically investigate differential impacts of intervention approaches on adolescents’ self-regulation behavioral trajectories.

Second, this study explored intervention effects solely through behavioral data, failing to uncover differential underlying mechanisms between the Embodied and Cognitive Task Groups at the physiological level. Future research could integrate multimodal physiological data such as Computational Rhinology to quantify airflow dynamics during respiratory interventions and investigate micro-level changes; functional near-infrared spectroscopy (fNIRS), and heart rate variability (HRV) monitoring to explore neurophysiological mechanisms; and extension to diverse daily life scenarios to examine generalizable mind–body interaction patterns (Johnsen, 2024; Farrokh et al., 2024; Zelano et al., 2016).

Third, this study’s generalizability may be limited due to its single educational stage sample (senior vocational high school freshmen). Future research could expand the sample to include multiple educational stages (e.g., junior high school, general high school, university) and diverse cultural contexts to systematically examine the applicability boundaries of embodied interventions across different populations. Moreover, incorporating multi-group validation across schools with varying educational resources and regional characteristics would help clarify the conditions under which breathing exercises and cognitive interventions are most effective.

Fourth, this study did not explicitly explore potential expectancy and placebo effects. Particularly in the Embodied Task Group, the novel breathing exercises might have induced positive expectations and placebo effects among participants. Similarly, the cognitive intervention involving daily diaries could have generated similar effects through regular self-reflection. The Mental Health Course Group likely lacked such novelty-driven effects, resulting in an imbalance of participants’ expectations across groups. For future research, it is recommended to adopt double-blind designs or incorporate a placebo control group (such as sham breathing exercises) to isolate the specific effects of the interventions from expectancy effects.

Fifth, although the study considered gender as a covariate in Experiment 1, the significant gender imbalance between the Embodied Task Group and the Cognitive Task Group could have affected the results of Experiment 2. Given that males and females may respond differently to interventions, future research should ensure consistent handling of gender as a variable.

## 5. Conclusions

Embodied interventions enhance the flow experience in the dimensions of “Unambiguous Feedback” and “Transformation of Time” through real-time physical feedback and multisensory integration. Longitudinal analysis further shows that embodied practices can guide individuals to shift from psychological evaluations to an awareness of dynamic mind–body processes and environmental interactions, forming a “body–environment coupling” cognitive framework.

Although this study is limited by the 20-day intervention cycle, the limitations of sample selection, and the use of purely behavioral data, future research can verify the long-term effects and mechanisms of action of embodied interventions through long-term follow-up, multimodal physiological indicators, and cross-cultural samples.

## Figures and Tables

**Table 1:** MANCOVA of intervention and pre-post test (Covariate: Gender).

Dependent Variable	Mean Square	*df_1_*	*df_2_*	*F*	*p*-Value	$\eta_{p}^{2}$
Unambiguous Feedback						
Intercept	403.30	1	439	486.56	<0.001 *	0.53 +
Pretest and posttest	1.97	1	439	2.38	0.12	0.01
Intervention	0.31	2	439	0.37	0.69	0.00
Pretest and posttest × intervention	2.64	2	440	3.19	0.04 *	0.01 +
Transformation of Time						
Intercept	274.92	1	439	290.53	<0.001 *	0.40 +
Pretest and posttest	0.63	1	439	0.67	0.41	0.00
Intervention	3.05	2	439	3.20	0.04 *	0.01 +
Pretest and posttest × intervention	1.05	2	439	1.10	0.33	0.01

Notes: * *p*-value < 0.05. + $\eta_{p}^{2}$ > 0.01.

## Data Availability

The datasets generated and analyzed during the current study are available from the corresponding author on reasonable request.

## Supplementary Materials

Relevant materials, such as the course PowerPoint and the audio for breathing guidance, can be downloaded from the website https://doi.org/10.17605/OSF.IO/T83AP.

## Abbreviations

The following abbreviations are used in this manuscript:

MANCOVA	Multivariate Analysis of Covariance
ENA	Epistemic Network Analysis
FB	Focus on Body
FPEF	Focus on Psychological Experiences and Feelings
FPPMC	Focus on the Process of Physical and Mental Changes
CPAS	Coping Process in Adverse Situations
PMSAS	Physical and Mental States in Adverse Situations
MFE	Mark Focus Events
ESEF	Extensive Situation Experience Feedback
PMSPE	Physical and Mental States in the Physical Environment
OC	Outcome Consciousness
PC	Process Consciousness
